# “The children of the Sun and Moon are the gardens”—How people, plants, and a living Sun shape life on Tanna, Vanuatu

**DOI:** 10.1371/journal.pone.0313997

**Published:** 2024-11-20

**Authors:** Michael J. Balick, Dominik M. Ramík, Nadine Ramík, Iahwa Kausas Nemisa Kumas, Gregory M Plunkett, Neal Kelso, Presley Dovo, K. David Harrison

**Affiliations:** 1 Center for Plants, People and Culture, New York Botanical Garden, Bronx, NY, United States of America; 2 Independent scholar, Lowanatom, Tanna, Vanuatu; 3 *Ieni Kausas* and *Tupunus* of Lamanamileg, Tanna, Vanuatu; 4 Independent scholar, San Francisco, CA, United States of America; 5 Vanuatu Department of Forests, Port Vila, Vanuatu; 6 VinUniversity, Hanoi, Vietnam; Tel Aviv university, ISRAEL

## Abstract

Based on original ethnographic and ethnobotanical research, we share how in the cosmology of Tanna, an island in Vanuatu’s southernmost province of Tafea, the Sun is viewed as a living, interactive being. Our initial interviews explored knowledge and beliefs concerning individual plant species, then subsequent follow-up interviews further explored topics that emerged therefrom. The results of these interviews are a series of oral narratives of the mytho-historical past involving the Sun, and the description of contemporary practices which are influenced by the Sun. In traditional narratives, the Sun is both a creative and destructive force which is sometimes viewed as an active, personified character, and in other circumstances appears as an instrument created and utilized by greater powers. People from Tanna recount—and we adopt as a hypothesis—that the Sun’s physical manifestation and role in the world has changed since the earliest days of its mythological creation, and that it remains an active player in Tanna’s biocultural landscape within practices including time-reckoning, agriculture, and architecture. Through its relationships with humans and non-humans alike, the Sun ultimately shapes the cultural practices and even the landscape of Tanna. The nature of these relationships is changing as linguistic and cultural practices shift alongside people’s relationship with the land, but the Sun remains a critical factor in lives and livelihoods of Tanna today.

“It is the son of Sun and Moon, he is *tupunus*, who is there inside [of Iasur volcano] and who gives the things to eat, because the Sun gives its sunshine for the crops to grow and the Moon gives the rain to water them. The one who returned to the volcano, he is the [original] *tupunus*.”

   —*Kaha* Iahwa of Lamanamileg, Tanna, Vanuatu

## Introduction

The Sun is of critical importance to all life on Earth, providing health and energy to humans and non-humans alike. People, particularly those who belong to biodiversity-dependent cultures, generally recognize the importance of plants in their lives, understanding that the Sun is essential to the growth and survival of a living plant. Through photosynthesis, plants have the remarkable ability to use the Sun’s energy to convert carbon dioxide and water into glucose (giving off oxygen as a by-product). This glucose powers the production of a wide variety of primary and secondary metabolites essential to human health and nutrition. Indigenous histories and cosmologies, however, view both plants and the Sun as much more than mechanical nutrient producers. Across the Pacific, traditional stories and religions describe the origins, animacy, and importance of the Sun, and local knowledge systems interpret ways that the Sun and plants help to enable healthy and happy lives. Thus, annual solar and environmental rhythms guide human activities, ranging from agriculture to health to architecture. In this descriptive ethnographic study, we explore the ways in which interactions between the Sun, plants, animals, and humans shape the lives of the people of Tanna Island, in southern Vanuatu. Oral narratives by Indigenous elders recount the role of the Sun in the mytho-historical past, including during the creation of the world. Subsequently, we describe the influence of the Sun on three domains of daily life on Tanna: time-reckoning, agriculture, and architecture.

The Sun appears in legends and stories across the Pacific, in which one can see that this celestial body is viewed as an animate force and was integral in bringing about the world as these cultures know it. The Sun was often not alone in these times of creation, but rather affected and interacted with a variety of human and non-human beings to achieve the feats in these stories. Thus, these diverse Indigenous cosmologies frame the Sun as a living, interactive person, who often remains as such in the present day. This latter point is evidence by contemporary cultural practices and narratives, which follow the stories referenced herein.

In Tikopia, a Polynesian outlier culture in the Solomon Islands, the octopus god Feke is closely identified with the Sun, with his arms invoked as solar rays in ritual incantations [1]. In Sāmoa, the creator god Tangaloa brought forth the Sun, whose heat in turn was responsible for all animal life [2]. In many eastern Polynesian legends, as well as Sāmoa, humanity’s earliest days were very short and the nights were very long because the Sun raced across the sky as he pleased. The cultural hero Māui snared the Sun, slowing it down and bringing people days and nights of appropriate length for their work [3].

Things were quite different in the early legends of the Banks Islands in northern Vanuatu, where there had been a time that had no night—only bright, endless daylight. The cultural hero Qat heard that the Torres Islands to the north had night, and so journeyed there to learn how he himself might bring night to his home. Upon his return, he caused the Sun to start sinking into the west. His brothers were frightened at first, but after night fell, they were finally able to rest [4].

In northern Vanuatu today, Loh islanders ensure that the Sun continues on its proper path following the summer solstice, in an annual ritual involving *Pandanus* baskets, magical chanting, and *palolo* marine worms [5]. At the other extreme of the archipelago to the south, in Vanuatu’s Tafea Province, the Sun plays a key role in what Netwar speakers call *temahwa* (*tamafa* in Nafe). This ritualized spitting of kava (*Piper methysticum* G. Forst.) connects practitioners to the ancestral spirits [6]. *Temahwa* is an important ritual on both Tanna and Aneityum islands, but on Aneityum, the Sun was worshiped regularly as a god known as Nagesega Rada. Aneityum’s Sun worship and magical practices were facilitated by the island’s hereditary paramount chiefs and by a caste of men who had the role of “holy priests.” On Tanna, no such organized worship of the Sun has been recorded, but it appears in local stories even today. Many of the stories are carried by the ‘special workers’ called *tupunus* in Netwar or variations in other Tanna languages (*tupanas* in Nafe ~ *tɨpunɨs* in Naka), who inherit powers to influence the Sun, weather, and specific crops (e.g., yams, *Dioscorea* spp., and taro, *Colocasia esculenta* (L.) Schott) [7].

In other contemporary practices, Pacific Islanders utilize their knowledge of the Sun to support themselves. These practices include detailed naming of the parts of the day and tracking the yearly progress of the Sun across seasons. Marquesans (French Polynesia) divide the day and night into at least nine named periods [8], as do the people of Sikaiana Atoll (Solomon Islands) [9]. Dividing the intervals of day and night narrowly, together with closely observing seasonal changes, tides, and fish behavior, are critical skills for fishermen in the Solomon Islands [10]. On Kiriwina Island, in Papua New Guinea (PNG), “star-gazers” keep careful track of the movement of asterisms in relation to the Sun and Moon, and relate these observations to their agricultural calendar [11]. Detailed methods for tracking the progress of the seasons are recorded for many Pacific cultures, including Sāmoan [12], Hawaiʻian [13], Chuuk [14], and Malekulan [15], and all ultimately guide the lives and livelihoods of these peoples.

The Sun can also influence the location and layout of houses, villages, gardens, and graves. On Muyuw island in PNG, people “believe that to have good garden results… houses must be built so that the Sun ‘jumps over’ a house’s central ridge, villages must be set out in (preferably) two straight lines running from east to west, yam houses must be set so that the Sun follows their central ridge, and gardens must *kikun kalas*, ‘follow the Sun’ the Creator’s path” [16:119]. Māori also oriented their houses to face the rising Sun, which according to Teone Taare Tikao [17] was to prevent *wairua* (ghosts) and *atua* (demons) from entering. Cultures across the Pacific are noted for orienting their dead towards the rising Sun, including Tongans [18] and Wogeo islanders in PNG [19]. The solar or astronomical alignment of many Pacific archeological sites are disputed, but oral narratives suggest that Odalmelech, the god-king of Ngermelech in Palau, may have ordered a series of stone faces to be oriented towards the rising Sun [20].

There is a longstanding centrality of the Sun in cultures of the Pacific, both in the distant past and the present. On Tanna Island, in southern Vanuatu, the Sun plays major roles in traditional stories and also informs a range of present practices, including several of those described above. In Vanuatu, all of these stories and practices fall under the concept of *kastom*, which Lindstrom describes as “festivals, along with any traditional or local practice, style, or belief” [21:5].

In pursuing this research, we find inspiration in Cynthia Fowler’s work [22] on the multispecies ethnography (or biosocial theory) of Sumba, Indonesia. Multispecies ethnography represents a current trend in anthropology that “centers on how a multitude of organisms’ livelihoods shape and are shaped by political, economic, and cultural forces” [23:545]. Fowler extends the discussion of multispecies relationships to all entities endowed with life in local ontologies, even if they are not included in the classification of life in Western science. As Kodi people on Sumba, Indonesia interact with what they consider to be living celestial bodies today, so too do the people of Tanna carry out their lives through regular interaction with stones, spirits, the Sun, and the Moon. Human interpretations of reality are shaped by interspecies relationships [24], just as they are shaped by relationships with entities outside of or beyond the biological concept of “species” [25]. In the present work, we aim to elaborate on how Tannese people have relationships with plants and a Sun viewed as living and interactive, shaped in the mytho-historical past and supporting healthy and productive lives today.

### Research questions

While conducting transdisciplinary fieldwork and investigating Indigenous beliefs on the Sun and daylight, we developed several research questions based on our initial conversations with local experts: 1) How does the Sun influence the activities and livelihoods of people on Tanna Island? 2) What agency is ascribed to the Sun in local tradition? 3) What do stories of creation and other mythical events reveal about how people perceive of the Sun in Tannese culture? 4) Are there separate domains of knowledge that are influenced by beliefs concerning the Sun?

A further goal of the present work is to afford Indigenous science communication with the same status as academic science communication. Our work is inherently transdisciplinary (anthropology, botany, and linguistics), involving collaboration with local knowledge partners who have very different methodological and storytelling strategies. We extend Turin’s observation that “collaboration works both ways” [26:855], ensuring that our work is not only mutually beneficial but that these plural methodologies are given equal footing. Thus, traditional stories, called *nagé* in the Netwar language of Tanna, make up a significant portion of the present text. We integrate these stories with our academic writing following Daigle et al. [27], who affirm that “storytelling continues to remain relevant as a way to connect the generations and for continued adaptation to ecosystem change and sustaining traditions” (p. 777). This is as true of Tanna as it is anywhere else, as the island “offers a rich and profuse narrative archive and Islanders keep this vital with recurrent retellings of Tanna’s times” [21:xiii]. The oral inventory of Tanna includes both contemporary storytelling, as in Lindstrom [21], and those stories concerned with the very origins of the world, as retold herein. Following the *nagé*, we discuss several examples of contemporary practices on Tanna that are influenced significantly by interaction with the Sun: divisions of the day, methods for tracking the Sun’s yearly progress, garden orientation, and house structure and orientation.

## Methods

### Study site

**Fig 1:**
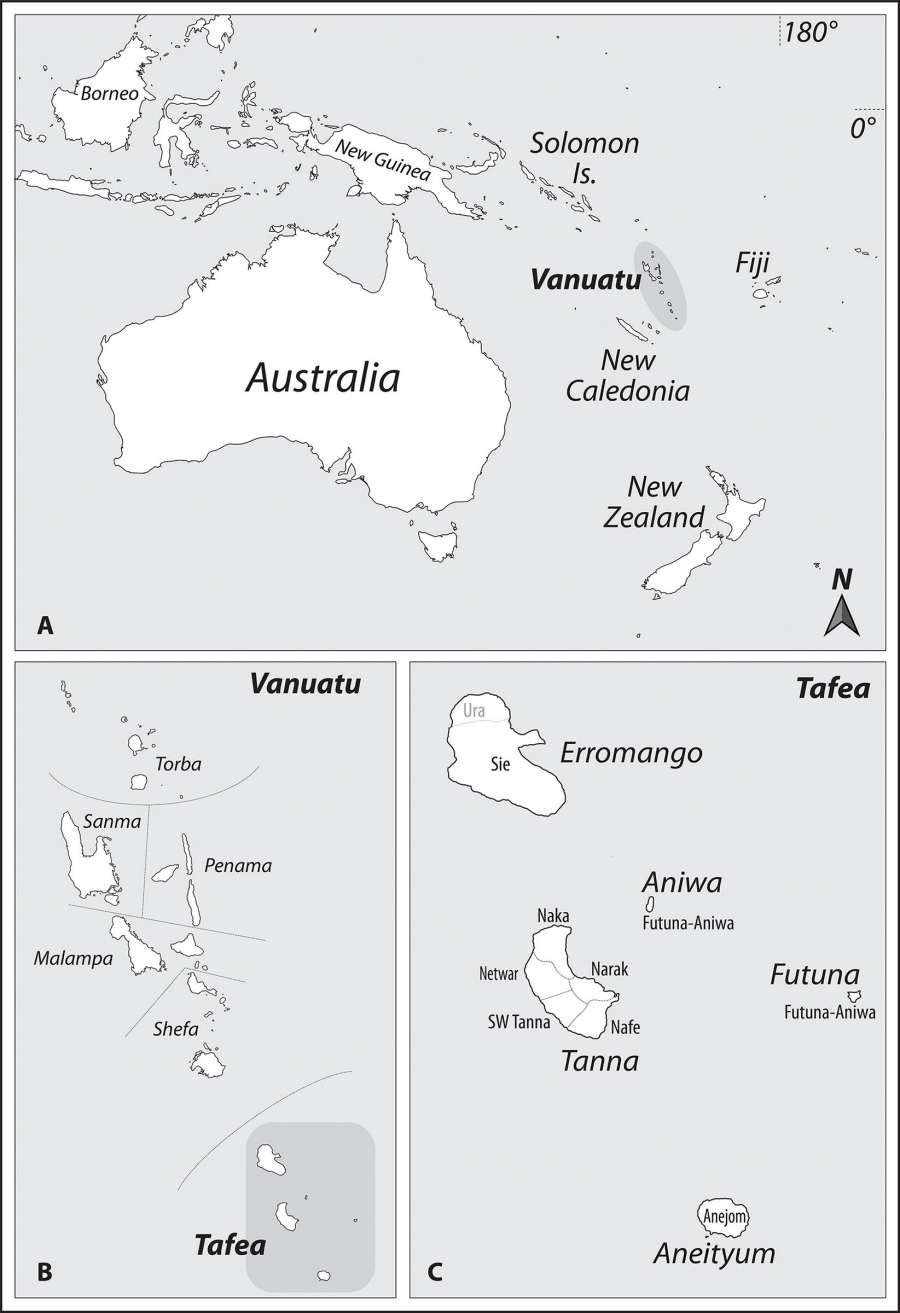
Map situating Tanna Island. A. Map of the southwest Pacific, highlighting Vanuatu. B. Map of Vanuatu, divided by province, with Tafea highlighted. C. Map of Tafea province, showing the five major islands of Erromango, Aniwa, Futuna, Tanna, and Aneityum, with approximate linguistic boundaries. Adapted from Oceania laea location map.svg by Uwe Dedering, under the Creative Commons Attribution-Share Alike 3.0 Unported license, and accessed https://commons.wikimedia.org/wiki/File:Oceania_laea_location_map_(%E2%88%92Hawaii).svg.

Vanuatu is an archipelago of more than 80 islands—65 of which are inhabited—mostly of volcanic origin. It lies in the central-eastern region of Melanesia, roughly equidistant between the Solomon Islands, Fiji, and New Caledonia (Fig 1). An estimated 138 local languages are spoken in the country by a population of around 300,000, meaning that Vanuatu is a global language hotspot, with the highest number of languages per capita in the world [28–30]. Five languages are indigenous to Tanna: Netwar (Lenakel), with 11,500 speakers; Narak (Whitesands), with 7,500 speakers; Neuai (and associated dialects in Southwest Tanna), with 4,500 speakers; Naka (North Tanna), with 5,000 speakers; and Nafe (Kwamera), with 3,500 speakers [31]. Nanunata, which also appears in the present work, is considered locally to be a separate language, but has not been listed as such in the literature.

Vanuatu is also situated within the East Melanesian Islands Biodiversity Hotspot [32]. Within Vanuatu, Tafea, consisting of the islands of Tanna, Aneityum, Erromango, Futuna and Aniwa, is the most biodiverse province, and these five islands are home to roughly half of the plant species found across the entire country [33].

### Data collection

We conducted ethnobotanical and ethnographic interviews on Tanna in 2016, 2018, 2019, and 2020–2023 as part of the *Plants and People of Vanuatu* project. Permissions for this work were granted in a Memorandum of Understanding with the Department of Forestry as well as a Research License granted jointly by the Vanuatu Department of Environmental Protection and Conservation, the Vanuatu Forestry Department, and the Vanuatu Cultural Centre. In individual communities, where the land is still under traditional tenure, permissions for plant collection and sharing the results of interviews were granted through written and oral prior informed consent processes. Our approach is not one of data extraction, but rather co-production of knowledge, based on mutual trust and reciprocity and the willingness of community experts to share their knowledge with us and through us to a global audience. The knowledge is shared with the understanding that it remains the intellectual property of its communities, and will be fully attributed to them, both individually and collectively.

Ethnobotanical interviews were conducted in English, Bislama, and local languages during or following the collection of plant specimens, when community members gathered to provide local names, general uses, and other lore about plants known to their communities. After taking notes on these data, each numbered plant specimen was prepared as an herbarium voucher and subsequently deposited at the Vanuatu National Herbarium (PVNH), with duplicates sent to the New York Botanical Garden (NY), the South Pacific Regional Herbarium (SUVA), and other international institutions. From the notes gathered during these initial ethnobotanical interviews, we identified several areas of interest, including the present topic, and conducted follow-up ethnographic semi-structured and informal interviews with about 20 consultants of varying ages, though mainly elders.

During these follow-up interviews, we discussed various open-ended topics relating to the Sun and daylight. In our prior informed consent process, we ensured that the individuals and communities sharing their knowledge with us knew to share only general knowledge—nothing considered to be a secret of the individual or their family. These follow-up interviews were conducted in Bislama, English, and local languages. After the ethnographic interviews we elicited more formal and ritualized tellings of traditional stories in several local languages of Tanna (mainly Netwar), which we audio recorded. Audio recordings were transcribed first into local languages, then translated to English. These appear in the present text. Unless noted as another language, words and stories herein are Netwar.

The program’s research protocols and methodologies were evaluated in 2015 by the Swarthmore College Institutional Review Board, which determined that research on this project does not constitute research with human subjects and did not require IRB approval. We obtained informed prior written and/or verbal consent for their participation from all cultural consultants.

## The Sun’s role in shaping livelihoods in southern Vanuatu

### The role of the Sun in Tanna’s past as recorded in *nagé*

In the earliest days of the world, or *nemoptan* (‘land, ground’ in the Netwar language), the powerful *nanemen* (‘spirit’) known as Wughin was responsible for many acts of creation. Iahwa, of Lamanamileg, and Katmatem, of Lamenahura, who are both Netwar-speaking *kaha* (‘elders, grandfathers’) from west Tanna, recount Wughin’s early actions as part of the cycle of *nagé* (‘stories, legends’) concerning the origin of the world and the things in it. There is some overlap in the accounts of these two elders, which were recorded separately, but together they represent the early history of the world as told through Tannese legend. After Wughin created the land, Iahwa and Katmatem describe his efforts to provide the world with light, and go on to tell of several actions by the hero figure Kalpapen.

In the beginning of the ground, there was a *niko* [canoe] that floated around. The *niko* was a [piece of] ground, it was the *niko* of Wughin and contained all his tools. When he wants to go somewhere, he takes it to go there. When he is done, he will fold it and put it ready close to himself. But he did not travel for nothing. He was traveling to create things. Before, there was nothing. No ground, it was just *nemagoago napnapen am* [lit. “the emptiness without interest”]…

   —*Kaha* Iahwa

Back in that time, it was the time of stones. One could not tell where the stone was and where the man was. That was the beginning. Wughin talked to that *kopiél* [stone] down here, who is our *kaha* [ancestor], to make this ground. He made from himself this ground here until he finished it, then he made plants and animals. The fowl, the pig, all the animals. They all came from within him.

**Fig 2:**
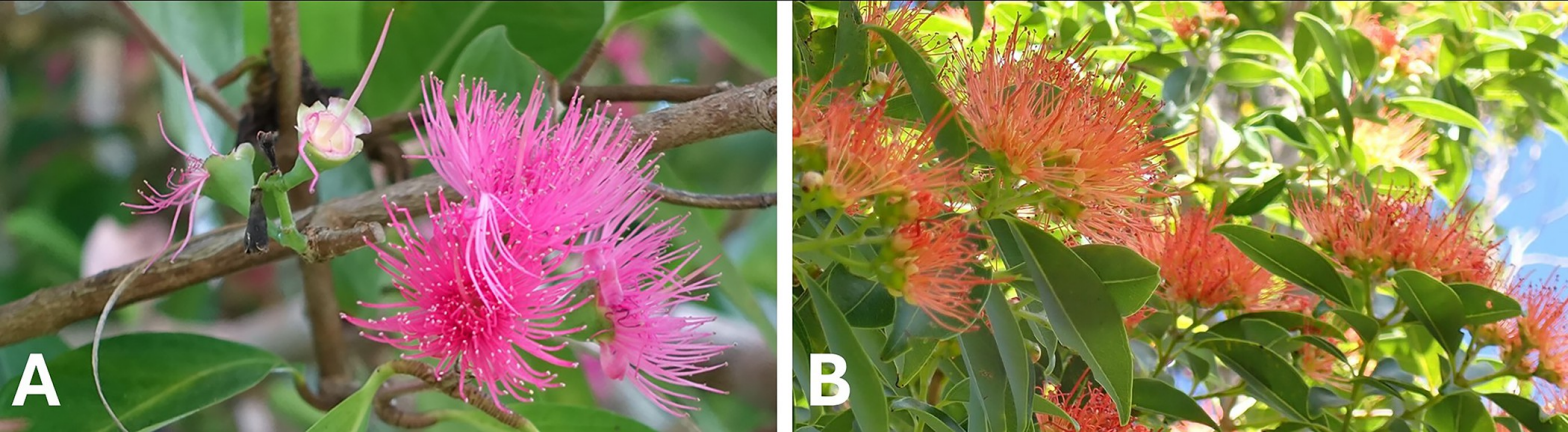
Wughin’s light-giving trees. A: *Nekawuk* (*Syzygium malaccense*). Photo by Michael J. Balick. Right: *Nekiléw* (*Metrosideros vitiensis*). Photo by Dominik M. Ramík.

He finished making all those things, but he saw it was still dark. So, he made a thing. He made [*nekawuk* trees, *Syzygium malaccense* (L.) Merr. and L.M. Perry] to shed light in the darkness [Fig 2]. Those trees made flowers to shine on the people [living beings] he made. When he made those trees and they gave their flowers, he realized that the shining they made was not enough to illuminate all those people he created. Thus, he made Mawuk, the Moon. He let it out of his hand and it flew up. It flew all the way up and shone there, but its light was not enough.

He thus made Met, the Sun. He let it loose from his hand and it flew up. It shone well, but illuminated only Niko Kapakol. That was a [stone-man]. But the stones, they are down underneath the ground, and Met did not shine to them. Met shone to Niko Kapakol, shone, shone, until Met lost his strength and was tired. So, he asked Mawuk to help him. And so now Met shines in the day, and Mawuk in the night. A *kaha* made it, *kaha* Léai [the traditional ancestor of this area].

—*Kaha* Katmatem

This story of *nekawuk* as a first attempt to give the world light is an important one. Other Netwar speakers provided additional detail on this part of the story, stating that the tree shines on the *kastom* roads of the *tupunus* and illuminates the road for him to obtain a good crop. Similarly, a closely related tree, *nekiléw* [*Metrosideros vitiensis* (A. Gray) Villon, Fig 2] is the light of *ierames* (‘spirits’) who work in the garden. This *tupunus*-*ierames*/human-spirit dichotomy in the stories of the light-bearing trees is a key element of the *nagé*. It foreshadows the splitting of the stone-men (powerful, magical beings who are the ancestors of all living things on Tanna) into their spiritual and mundane halves, an event that does not actually occur until Kalpapen brings into being the day-night cycle that we know today. This further suggests that even though the period when the trees provided the primary light of the world is over, these trees still give off light to the *tupunus* and spirits today, after the splitting of the stone-men.

Katmaten’s account is also a shortened telling of events. There was actually a long period of time between when Wughin created Mawuk and Met and when the world saw the first cycle of night and day, as we have today. In some retellings of the *nagé*, this period was one of eternal day—light that bore down constantly on the people and would not allow them to rest. In Iahwa’s telling, there were a series of eternal days and eternal nights, a period of “all the time night and all the time day,” after which Kalpapen set the Sun on its current path.

When Wughin finished all this and gave to Nias Apen [a stone-man] *noukaren narekan* [lit. “the root of life;” the *kastom*] and gave him the winds, he put Met to its place to walk there. Until that time Met was there without movement. That is because in the beginning it was all the time night, then it was all the time day. During the time when Kalpapen and Pwita were breaking off the ground with their feet, they were also putting things to those grounds, all things that the ground needed by order of Wughin. It was then, when it was all the time day and all the time night, because Kalpapen did not yet drink his kava. At that time, they were only drinking *nakwiam* [less potent wild kava, *Piper wichmannii* C. DC.]. Kalpapen then came with the real kava [*Piper methysticum* G. Forst.] and went on breaking the ground as Wughin told him. When he puts his foot on a ground to break it, here comes the day, when he puts another foot on another piece of ground to break it, here comes the night.

When it is an eternal day, people go to make their gardens. When they are tired, they drink *nakwiam* and make *temahwa* (ritual spitting of kava) so that night comes, but nothing happens, so they have a short rest during the day and then go back to the garden.

Then Kalpapen went to Lownekiamapen as the “eternal night” was about to fall. He brought the kava and a black rooster, telling the rooster to announce the day when it comes. When the day came, the rooster called, seeing Met appearing. Kalpapen untied his kava and made a *temahwa* to make night again. Performing this *temahwa*, he untied the rooster to announce from now on forever the coming day. Then he untied the kava root so it is used for *temahwa* with falling night. Then he untied all the stone-men. There came out men to walk in the daylight and the [immobile] stones to walk in the night. Kalpapen made only one such *temahwa*, but ever since this *temahwa* is repeated by men. When Kalpapen made his *temahwa* ready, he summoned all the lands to take part in his *temahwa*. When the *temahwa* was done, Kalpapen untied all those things, the night was coming and *iawitaleg* [cicada] started to sing.

So, Met walked towards the horizon and many *iawitaleg* sang. When they were singing, it was already the falling night, and the stones, who separated from stone-men, were frightened. They walked towards the night and the men, who came out from those stones, and went to sleep. Met went on until disappearing altogether beyond the horizon. Men slept and Met walked on to return on the other side and when rising again, men woke up. At this time, the stones, who were walking during the night, went to sleep. Mawuk also went to sleep as she is also a stone. She went during the day to do her work elsewhere out of sight. From this time on Mawuk works in two places. Sometimes she works in this world and sometimes she goes somewhere else to pursue her work during the day, but it is not here.

As it was decided so, now men perform their *temahwa* towards the stones [out from which they came] who remained living in the night. Kalpapen put all into its place, the day, the night, and the kava between the day and the night. So, before that, all walked together, all were men [stone-men], the plants, the animals, the men… Kalpapen divided the living beings [the stone-men] and put for example the stones [the stone half of the stone-men] of *nawuk* [a plant, *Inocarpus fagifer* (Parkinson) Fosberg] aside and the *nawuk* [the “actual” plant] aside. He puts the stone of breadfruit [*Artocarpus altilis* (Parkinson) Fosberg] aside and the breadfruit [actual plant] aside. Each plant giving food was separated from its stone. He then placed the stones into different *nakamals* [ceremonial gathering places]. It is those plants, which give food, that Kalpapen summoned and made *niel* [ceremony of crop sharing] with them.

—*Kaha* Iahwa

In this section of the *nagé*, Kalpapen becomes the major actor—under directions from Wughin. Kalpapen is responsible for setting the present cycle of night and day. A group of elders from the village of Enfitana (in the Nahual language area) explain that Kalpapen set his rooster to send off the Sun every evening with the call “*tata naméwén*” (father, you go), and then at dawn to call again “*tata nawéhé*” (father, you come). More importantly, Kalpapen performed the first *temahwa* ritual as the Sun set on the eternal cycle. The kava drunk for a *temahwa* is *tapen*, the door, between day and night, between the world of the living and the world of spirits. When Kalpapen makes his *temahwa*, the day ends and the night begins. Simultaneously, the stone-men that represent food plants split into their stone halves and their plant halves. The living plants now inhabit the daylight with men, while the living essences of the stone-men are active at night. Today, *temahwa* is still performed as the Sun sets, so that the Sun can carry humanity’s requests to the realm of the spirits that inhabit the night.

Two Nahual-speaking elders, Tom Kota of Imapul and Masi of Ienpenhawan, provided a somewhat different story for the reason the Sun follows its current path, grounded in their local geography in southwest Tanna. In the olden times, when there was everlasting day, Kalpapen brought the first kava and made the first *temahwa*, causing the coming of the night. He made the Sun, which was created by Kugen [Wughin] in time beyond memory and insofar as it was fixed straight overhead, to move towards the west and down into the water. He achieved this by planting an enormous *tabu* stone, indeed a mountain, called Tukos Alal (lit. ‘Mountain that Revolves’) in the depression just between Tukosmera and Melen. As Tukos Alal rotates, it makes the Sun turn around Tanna and this ensures that after the first night of Kalpapen, the Sun would come up again and bring a new day, and it does so until now. When visiting the mountain, which is hard to find, one should cut marks on trees as one progresses towards the mountain. At one point, the visitor will see his own marks in front of himself, instead of behind him as he advances, as the mountain turns everything around. Then he knows he is on the top of the mountain.

When Kalpapen caused the first night to come, it was pitch dark and without light. Thus, the stone-men made a large *nowanuman* ‘earth oven’ and heated its stones until they were glowing. They then took the stones one by one with *kuhwa* ‘wooden tongs’ and put them to the sky to shine there. The small stones made stars, but the big stone called *iaseknal* was put much closer and became the Moon. The Moon is the closest of the celestial bodies, a little behind are the stars, and far, far behind these two is the Sun, which is a very powerful fire—if it were closer than the stars, it would scorch the Earth. This story is not secret knowledge, but it is (according to Tom and Masi) known only to the people of Imapul and up along the *kastom* road towards Tukosmera. They said, not without pride, that even the people in other countries are not aware of the fact that it is a mountain set by Kalpapen next to Tukosmera that causes the Sun and the entire sky to revolve.

*Kaha* Iahwa’s recitation of the *nagé* origins cycle continues (in Netwar) with the story of *Tagalua*, a marine snake (banded sea krait, *Laticauda colubrina*). After this follows the story of the first *tupunus*, who is the son of the Sun and Moon and a metaphorical analogue to the gardens themselves.

They buried [Tagalua, the banded sea krait] and kept the place clean and well swept, until one day a plant came out from the ground, from Tagalua’s eye. It came out from the grave which they swept every day. The plant grew bigger and bigger, it was a coconut tree [*Cocos nucifera* L.]. The other eye of Tagalua broke and there came *tehé* [the sea] out of it. His sons and his wife made a *nowankelu metkaparen* [a fence made of interwoven wild cane, *Miscanthus floridulus* (Labill.) Warb. ex K. Schum. & Lauterb.] around…

This is why the coconut can be planted wherever the strong Sun is. It has no problem with Sun because it grew well from Tagalua’s eye on a well-swept and sunburnt ground. But when you plant coconut trees in places cold and without sunshine, it may only give small fruit and not grow well…

—*Kaha* Iahwa

The location and layout of the gardens of Tanna are arranged based on topography and light exposure (to be discussed further in the next section). This *nagé* is an explanation of the preferred growing location of one of the island’s staple crops, the coconut. Coconut trees do much better under strong sunlight, particularly in coastal forests at the very edge of the beach. *Kaha* Iahwa’s next story introduces the son of the Sun and Moon, who is both the first *tupunus* and the origin of Tanna’s gardens.

Sometime later, someone—a child—came out from [Iasur volcano]. He came out and people killed him. They put him inside a cave and from there he came out and followed his path underground back to the volcano [as people are expected to do when they die]. The Sun and the Moon came there, because he was their son and they shined into the caldera of the volcano. They were looking for their son. Their shining into the volcano, it was their weeping—they were mourning their child. Inside the volcano, there are different vents, that is where different food comes from [cooked as in an earth oven]. If you call there to one of the vents to have a fruit or a crop, you will have it, because it is the *tupunus*, each vent gives something. It is the son of the Sun and Moon, he is *tupunus*, who is there inside and who gives the things to eat, because Sun gives its sunshine for the crops to grow and the Moon gives the rain to water them. The one who returned to the volcano [the son of the Sun and Moon], he is the [original] *tupunus*. This man came out to speak to people of *narekan min* [the “right ways of life,” the *kastom*], but people did not want to listen to him and they killed him. They put him into a cave and went to dig out a *tapuga* kava [chiefly kava grown in a *nuto* tree-fern trunk (referring to several species in the family Cyathaceae)].

Wughin, who is [the original] *ieramara*, sent a man to see where the child is. He wanted to see what happened to the child whom he sent to do work. But the kava the men dug out to drink is the one that only *ieramara* [Wughin] can drink. They did so as if they killed a *ieramara* with the kava that belongs to him. But the man sent by Wughin asked: “Why did you kill that one [the child of the Sun and Moon]?” But they were just sitting there not knowing what to say. He asked: “He came, what did he do wrong that you killed him?” They then went away and were drinking the kava in Lowtapuga. This is to say that *tapuga* is for the *ieramara*, but the low kava is for ordinary men. The man [who Wughin sent] asked them again, [but they were] speechless. This is why this place is called Léiawag [the place where people are open-mouthed and speechless].

—*Kaha* Iahwa

The Sun and Moon, as the most prominent and light-giving celestial objects in the sky, are responsible for the success of Tanna’s gardens, both through photosynthesis and the island’s traditions. In some traditional accounts, they are the mother and father of the gardens themselves. In this version, the child of the Sun and Moon is the first *tupunus*. Today’s *tupunus* are responsible for the health and success of gardens on Tanna, and as such, they stand in metaphorically for the gardens. Without the Sun and Moon, there are no *tupunus*, and without the *tupunus*, there are no gardens.

The following two stories are still origin stories in a sense, but do not recount the creation of the world. Instead, they involve the Sun as an active character and serve to explain how some things work in the present day. The first story involves the Sun as a malefic spirit, rather than a mythical force of creation, and teaches listeners about the role of the *tupunus* and some aspects of *kastom* exchange. The Sun also takes a role in the second story, which serves to explain in part the behavior of several species of lizard native to Tanna.

A [stone-man] lived called Mawil. He used his *niko* [canoe] to hit a *ierames* spirit called Nuwalauhia [lit. “yellow grass,” the Sun stone-man himself]. That Nuwalauhia destroyed the gardens of people. He burnt them, making the plants become yellow. It is like when today there is too much sun and all becomes sunburnt and yellow. He walked wherever he wanted. Wherever he went, plants became dry and died. It is so that Nuwilafil [another spirit] was asked by the people to help them to kill Nuwalauhia. But Nuwalauhia knew about that, so he came to devour Nuwilafil. As he approached, he saw Nuwilafil sleeping in his place on the other side of the creek in the *nakamal* of Lowneparu with his *niko* attached to his leg. Nuwalauhia sang a song, to see if Nuwilafil was indeed sleeping. Nuwalauhia crept closer, but he heard a song being sung in answer, and thinking Nuwilafil was awake, he left. But it was the voice of the *niko* that he heard. The *niko* woke up Nuwilafil, telling him “Wake up at once, because you nearly got devoured by Nuwalauhia. I saved you though.”

Nuwilafil got up and ran with his *niko* and his spear down towards the seashore. He went into his *niko* and paddled far out on the open ocean. There he waited. But before running away, he put at his sleeping place a wooden log. Nuwalauhia waited for some time and when he thought that Nuwilafil was finally asleep, he crept back. Seeing the log and thinking that Nuwilafil was indeed sleeping, he jumped on it and devoured it at once. But in his mouth, he felt it was a trunk of wood and he spat it out. That was the log that Nuwilafil put at his place. Nuwalauhia pulled wind into his nostrils in from the north, but he did not smell Nuwilafil. Then he pulled the wind from the west and south, but to no avail. Eventually he smelled towards the sea and indeed he smelled Nuwilafil. So, he ran to the sea, he smelled him far in the canoe. He threw himself into the sea, he jumped at Nuwilafil to devour him. But as he was jumping, Nuwilafil pierced him with his *suk* [a kind of spear tipped with a sharpened xylem of *nuto*, a species of tree fern of the family Cyatheaceae with a hard stem]. He speared him on and on until Nuwalauhia was dead. Then he took his body and put him on the sand beach. When Nuwalauhia died, all saw that the strength of the sunshine weakened and that rain began to fall down. That is why now, when we need to make rain, we make it [with stones], there is nothing impeding us anymore.

Now that Nuwalauhia was dead, Nuwilafil went to plant his new garden. He planted *nuwha* [island cabbage, *Abelmoschus manihot* (L.) Medik. and other leafy species], as well as *naru* [sugarcane, *Saccharum officinarum* L.]. He planted *nuwha nesiwan tuan* and *nuwha nesiwan apen* [lit. island cabbage with the white and with the black excrement of *hiuwan* snails, genus *Turbo*]. When it is the harvest time, he cuts the banana trees in his garden [to collect the bananas, which are species and hybrids of the genus *Musa*], and he takes the two kinds of *nuwha* (island cabbage). All people around him plant in the same way [suggesting he is the *tupunus*] and when harvest comes, they bring their crop to him. He also works on *hiuwan* [meaning he is *tupunus* of this kind of snail]. That is why during the harvest time people have to bring the snails to him to eat. They bring the two colors of *hiuwan* and exchange them with him against his two colors of island cabbage and bananas. Because it is him, who is in charge of *hiuwan*, it is he who eats it [before everyone else]. Those who bring *hiuwan*, they swap it with him.

Later, when it is time of *kamaru nuw* [blessing of new yams, *Dioscorea* spp.]—the evening before—Nuwilafil goes to his *ik awsim* [taboo place for *tupunus* to work his stones] and exclaims: “Tomorrow I will bless the yams” [so that the *ierames* of the yam stone hears it and knows his work is over]. He also goes down to the creek where the sacred stone of *nemankat* [sunshine] is and he exclaims the same. This was all then repeated by the people until today. The next day they will dig out the yams, cover with leaves of *nuig* [*Miscanthus floridulus* (Labill.) Warb. ex K. Schum. & Lauterb.] and attach with vines of *neparem* [*Pueraria montana* var. *lobata* (Wild.) Maesen & S.M. Almeida ex Sanjappa & Predeep]. Then they take a log of dead wood as the one Nuwalauhia ate and all go to the sea to throw it there for [Nuwalauhia] to remember what he has done. But this is not really done anymore, only I remember that.

—*Kaha* Iahwa

In this story, the Sun is a destructive force. Still a stone-man, named Nuwalauhia for grass yellowed by his heat, his power is too much and must be checked. After Nuwilafil kills the Sun, the power of the *tupunus* is greater. They are now able to summon rain, unhindered by the Sun’s heat. In reality, this death was not the end of the Sun—he is still in the sky. Rather, Nuwilafil merely weakened him. Nuwilafil goes on to become a *tupunus* himself, in charge of both yams and snails, and plants many of the staple crops of Tanna. During a time within *Kaha* Iahwa’s memory, a ritual recreating the struggle between Nuwalauhia and Nuwilafil was still practiced, in celebration of the weakening of the Sun’s strength. This story reveals a sense of balance in the mythology of Tanna; the Sun is an integral part of life, but can be destructive. Sometimes there has to be active work to reduce the negative effects of the Sun, such as shading crops, keeping track of calendar plants, or performing weather magic. The songs of Nuwalauhia and the *niko* are still known to *Kaha* Iahwa—he sang them during this telling—but they are in Nakharan Awas, the ‘ancient language’ that is no longer understood.

Following is another *nagé* translated from Netwar, recounted by *Kaha* Sauté from Lamanafa.

There was an old woman who lived here. Her name was Noram [lit. ‘your little sister], she had two boys. One was of black color, the other was of red color. They lived here and sometimes went down to the creek. There was a *ierames* [who is *kaliawen*, the shark] who lived down there, who was eating all the people on the ground [island] around. He ate all the people till he ate the red-skinned boy of the old woman. He ate the one with red skin and let go the one with black skin. He ate the red-skinned boy and his mother cried and was searching for her missing boy and tried hard to find out who can kill the *ierames*. It is because he ate her boy. She searched, searched, searched on till she found several *meliak* [a general term for lizards covering mostly skinks of the genus *Emoia*]. First, she saw *kapiarus* [the skink *Caledoniscincus atropunctatus* (Roux, 1913)]. *Kapiarus* is black, his skin is nicely smooth, full of energy. She sent him [to the *ierames*]. “Go there, if you can kill the *ierames*.” He went and saw the place where the *ierames* slept, he saw he indeed slept there. He sees how he breathes and when he exhales, leaves of trees fly away. He sees how the *ierames* breathes and the leaves fly so he sees he cannot even go close to him so he returns. He says “I went there, but I could not approach him so I came back.” So, she sends *napuram* [the gecko *Nactus pelagicus* (Girard, 1858)]. *Napuram* went closer and closer and went up to a tree. He climbs on the small branches and twigs close to the place where *ierames* sleeps thinking he will jump down to him, but he feels that the strength of the *ierames* is greater than his own. He remains up there and is frightened. He is so frightened it is like he is petrified and then he returns. So, she sends *iaru* [the skink *Emoia cyanogaster* (Lesson, 1830)], which we today call *iaru kaliawen*, because he chased away the *ierames kaliawen*.

So, they sent *iaru*. But *iaru* says he will go with *wulawula* [a bird, *Lalage leucopyga* (Gould, 1838)] and with the fire—fire, meaning the Sun, and *wulawua* is that bird. So *iaru* speaks to *wulawula* and Met [the Sun], “It is you who will kill his power [of the *ierames*] and you, *wulawula*, you will be on guard, when the fire [Sun] comes straight above, you tell me and I will throw myself at him.” So, he [*iaru*] went silently on till he was close to the *ierames* and he remained there hidden awaiting the signal of the two. So *iaru* is hidden [with the *ierames*] in the middle of a patch of *nuig* [wild cane, *Miscanthus floridulus*] and he moves the *nuig*. *Ierames* wakes up and sees something tickle him. While he does so, the Sun runs up [in the sky]. *Ierames* feels strong tickling and feels chills, thinking something is about to come upon him. So, he stays there lying with chills and feeling that the heat is increasing with the Sun going up, he indeed feels that the patch of *nuig* is very hot. Then *wulawula* called and the patch of *nuig* caught fire from the Sun. *Wulawula* calls, you know, during noon in this way–“tu-weet, tu-weet.” *Wulawula* calls and it caught fire, so *iaru* jumped down with the fire [Sun]. *Ierames* runs away. He stops to do something, but *iaru* bites him, the fire burns him, *iaru* jumps on him and he is frightened so he runs away. They chase him, chase on, he wants to turn, but *iaru* and Met chase him all the way down to the sea. They chase him until he sees a stone in front of him. He turns as the fire burns him and *iaru* attacks him, so he jumps and breaks the stone in the middle and hurls himself into the sea. *Iaru* tells him, “your place is in the sea, my place is up here.” Then *iaru* called up for his several *meliak* and told them to keep guard between the sea and the ground, so *kaliawen* [the *ierames*] would not return. “You, you will live here and keep guard of this place. If you see he comes back, you will send a word to me and I will come to strike him.”

So, they are still there, if you go there, you will see them [around that stone]. That is the sign ever since, now towards noon you will hear *wulawula* who sits on the top of the *nawula* [*Macaranga dioica* (G. Forst.) Mull. Arg.] tree and he calls. And for *iaru*, it is the same, we may be sitting around lunchtime talking and he falls on us. That is why we call him *iaru kaliawen*. And for *napuram*, you will now take in hand a *napuram*, you can try it, take a small branch, you put the *napuram* on the branch really low above the ground, you will wait for it to jump down, you can wait till the night, he will be too afraid to jump. He is afraid—it reminds him of the moments when he was above *kaliawen* and could not jump. So, the *nowanagé* [legend] is our story of our place. *Kaliawen* ate the red man, leaving the black man, that is us. But if he did not eat the red man, he would live with us till today. So, he took away the red-skinned man and left the black man. Now we have here white men too. If he [*kaliawen*] did not do that, we would have red men too here. That’s it.

—*Kaha* Sauté

**Fig 3:**
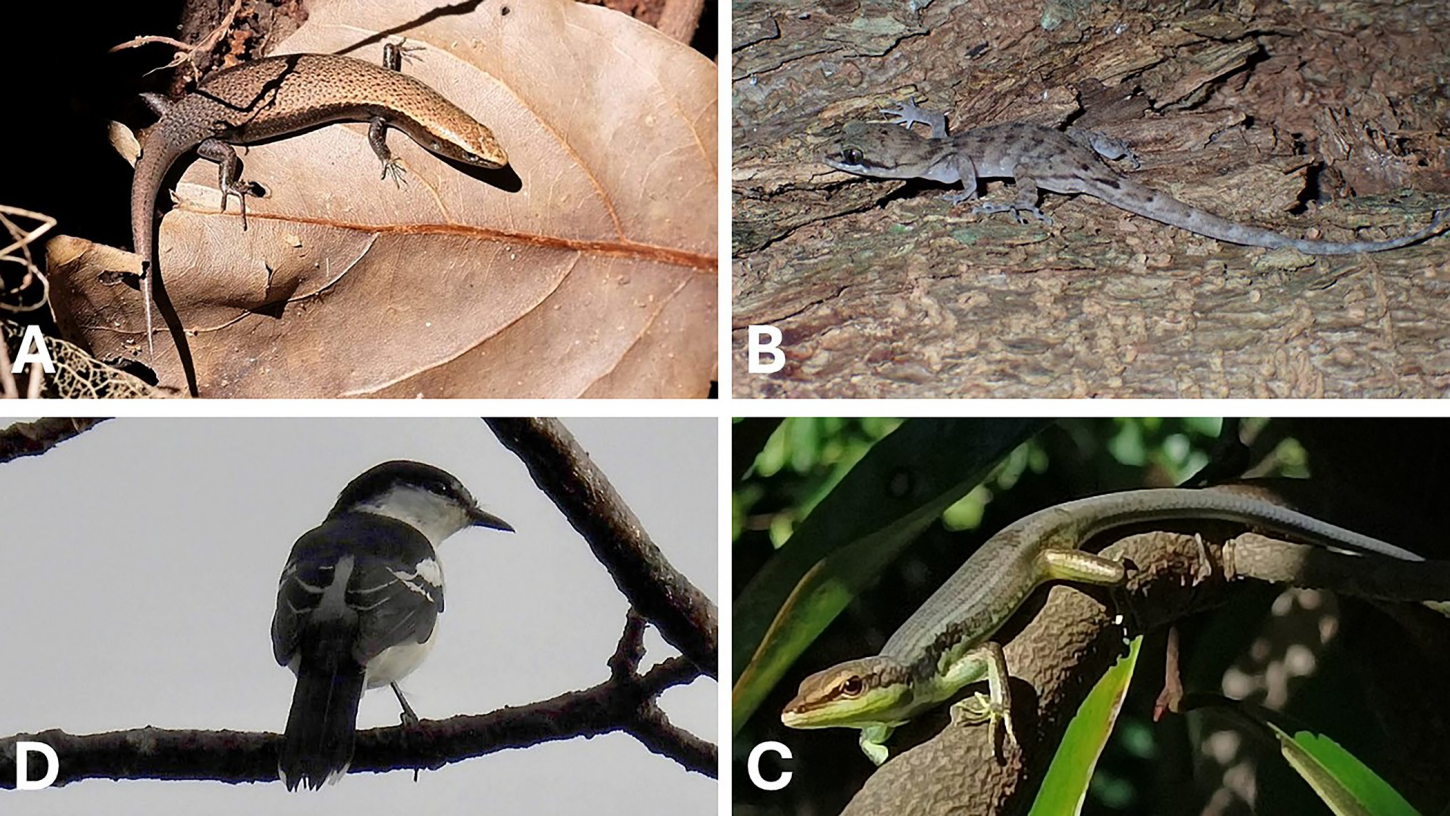
Animals from the shark spirit story. A: *kapiarus* [*Caledoniscincus atropunctatus*]. B: *napuram* [*Nactus pelagicus*]. C: *iaru* [*Emoia cyanogaster*]. D: *wulawula* [*Lalage leucopyga*]. Photos by Dominik M. Ramík.

Though the events of this story occur when these animals (Fig 3) are still stone-“men,” they serve primarily to pass on knowledge about the behavior of lizards and to explain why the shark’s place is in the sea. The lizard *kapiarus* is rarely, if ever, seen to climb trees, so he is not able to climb above *kaliawen* in the story. *Napuram* climbs, but won’t fall down on people, so he is quite harmless—he won’t frighten anyone by jumping on him. *Iaru kaliawen* is a very slender skink and often climbs the thinnest branches, and is thus also susceptible to fall down on people and frighten them. This is known to occur frequently around noon. The bird *wulawula*, just as it warned of the approach of the Sun in the story, today announces when the Sun is about to reappear after a rain. The Sun in this story is still powerful, able to burn away a malevolent *ierames*, but is a positive figure, in contrast to the previous story of Nuwalauhia. Lizards, birds, and the Sun all cooperate in the story to push the evil figure of the shark off of the land and keep him in the sea for the rest of time.

### The role of the Sun in livelihoods on Tanna today

Tanna’s traditional stories reveal important aspects of the place of the Sun in the islanders’ cosmology. By also looking at present-day practices, we can see the relationships that play out between people, plants, and the Sun today.

One such practice is the tradition of orienting graves “downhill” on the island’s west coast, so that the face of the deceased looks upon the rising Sun. Graves are also decorated with plantings of *narwiu* [*Cordyline fruticosa* (L.) A. Chev.], which is a symbol of everlasting life, conveying the idea that the dead may look upon the rising Sun for all time.

Other Sun-related practices that we do not discuss in detail here are medicines to mitigate negative effects of the Sun, such as sunstroke, and methods of magically influencing the Sun. Both of these topics often involve individual or family secrets, but some are general knowledge. For example, the following is widely known: When the Sun is moving “too quickly” towards the horizon and someone is running late, they can use magical plants such as *nuig* ‘wild cane’ (*Miscanthus floridulus*) or *nyhal taha Met* ‘grass of the Sun’ (*Paspalum* sp.) to stop the Sun in its path and grant them more time. The name *nougemet* refers to multiple species of grasses—including *Isachne comata* Munro ex Hack. and *Cyrtococcum oxyphyllum* (Hochst. ex Steud.) Stapf—which are also used to pause the Sun. An example of general knowledge relating to health can be found in calendar plants, some of which are indicators of the hot season and are thus signals informing people that they should work less as a precaution against heat-related illnesses. On Tanna, the presence of flower buds on *nuig* ‘wild cane’ (*M*. *floridulus*) indicates the hot season, while on nearby Aneityum, the browning of *inya* (*Casuarina equisetifolia* L.) signals the same.

The topics below cover several ways that people interact with and absorb knowledge from the Sun to facilitate their livelihoods. All are or were general knowledge, but some (such as the “living Stonehenge” and traditional house structure), have fallen out of widespread use in recent years.

#### Dividing the day

People on Tanna follow the progress of days both directly by the movement of the Sun and by signals given by the diurnal schedules of certain plants and animals. These signals provide cues, such as when to complete gardening, when to eat, and when to prepare and drink kava, among other things.

As is common in rural areas across the globe, one is generally awoken in the morning by the roosters’ calls. These animals were brought to Vanuatu on some of the earliest Austronesian settlements of the archipelago [34]. As roosters may also call somewhat randomly during the night, especially when it is cold or the Moon is full, one waits for the repeated and sustained rooster calls before getting up. In fact, locals recognize three separate kinds of rooster call, and not until the last is heard should one arise. The first kind of call is *menek rakawinen tat* (lit. ‘the fowl calls badly’) and is the kind of call heard any time during the night. The second is *menek rakawinen* (lit. ‘the fowl calls’), and may happen very early in the morning while the rooster is still up in the branches of his tree. The third is *menek rakawinen ut* (lit. ‘the fowl calls well’). The third call is when the Sun is about to rise and the rooster descends from his tree after the call.

**Fig 4:**
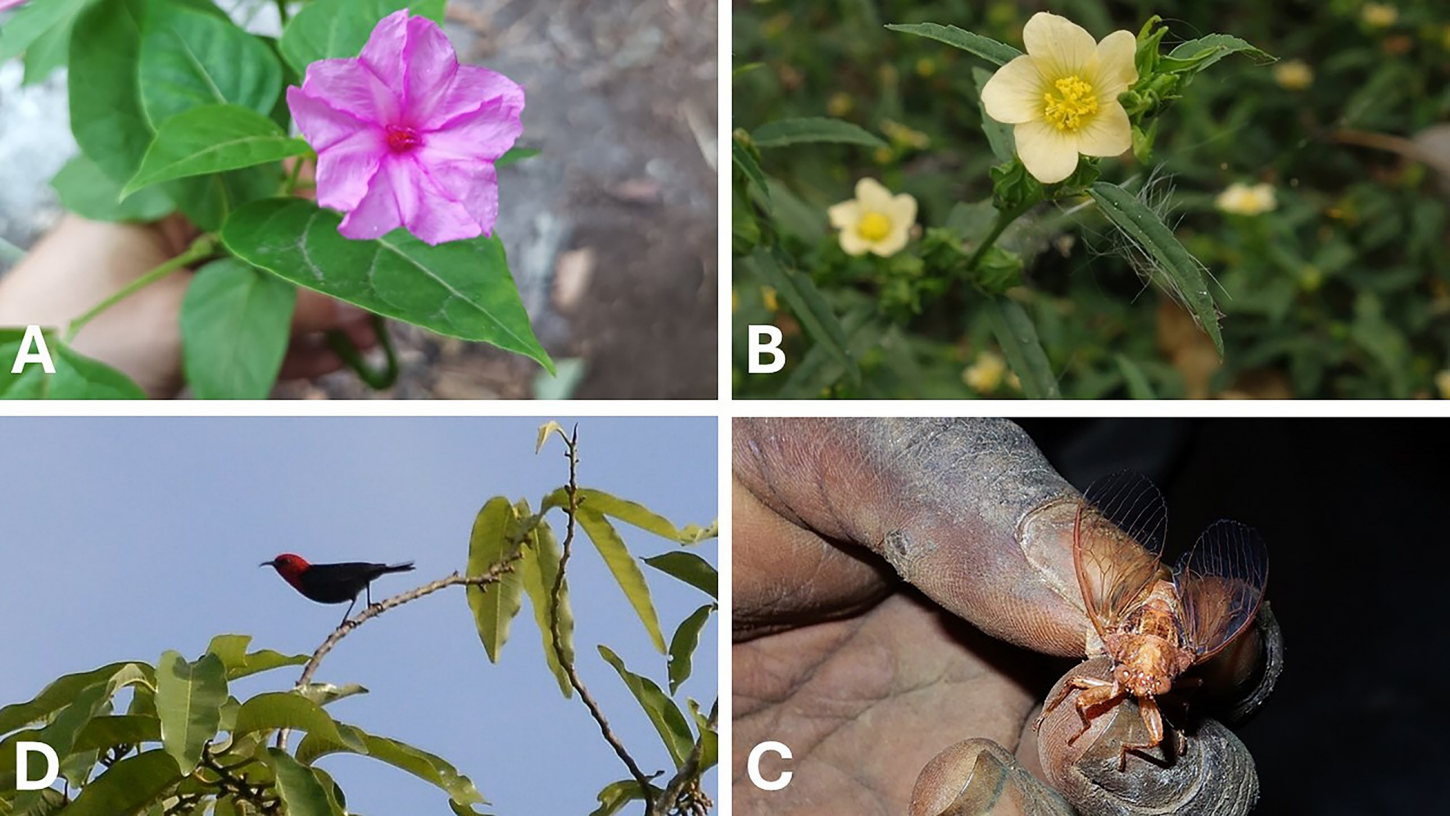
Netwar time-telling plants and animals. A: *nougement* (*Mirabilis jalapa*). B: *triklok* (genus *Sida*). C: *iawitaleg* (a cicada). D: *nakoam* (male *Myzomela cardinalis*). Photos by Dominik M. Ramík.

Newai speakers keep watch for the flowers of *kisep korién* (*Sida rhombifolia* L.) to open to know when it is time for lunch. An introduced plant, *nepegién* (*Mirabilis jalapa* L.), has been incorporated into the local time-keeping system—it flowers continuously throughout the year and elders are said to “use it as their watches.” They observe the flowers opening gradually in the early afternoon, and by the time it is half open, women should prepare *nahunu* (the food for after kava). When the flower is fully open, it is time to stop working in the garden and drink kava. To Netwar speakers, the end-of-work signal is cued not only by fully open flowers of *M*. *jalapa* (known to them as *nougemet*), but also by the opening of another flower, that of *triklok* [Bislama “three o’clock,” *Malvastrum coromandelianum* (L.) Garcke], as well as the song of *nakoam* [a bird more commonly called *kowiaméta* today, the male *Myzomela cardinalis* (Gmelin, 1788)], and by the singing of *iawitaleg* cicadas (see time-telling plants and animals in Fig 4).

In Southeast Tanna, in the Nafe language area, the Sun’s path for a single day is followed in reference to geographical features and ties into local cosmology and ritual. The Sun rises each day in the east. As it crosses the sky, it visits all the gardens of South Tanna. It then sets in the west, crossing a mountain pass called Kwarua, ‘doorway of the Sun,’ and sets at a place called Iwaru, which is the coastal area of southwest Tanna where there is a black sand beach and a large black stone in the ocean surf. As the Sun sets, it “stamps” the large rock with a “report” of all the gardens it visited (people report that they can feel the earth move as it stamps the rock). If the report is bad for any individual garden, then the crops will not grow well there. If a person’s garden is bad (not productive), then they know the report was bad (which probably means the gardener did something wrong). However, in such a case, the *tupanas* can perform a *temahwa* to make it rain and the garden owner can start a fresh garden. Elders Nepio and Loman of Lamanafa explain the *temahwa* of the *tupunus* as follows:

What a *tupunus* does, when he performs his work, is that in the evening, the time between day (dedicated to men) and night (dedicated to stones and spirits), he re-creates the first *temahwa* of Kalpapen and uses the kava and *temahwa* to enter again into close connection with the stone out from which he (his ancestors) once came. United again through kava with his stone self, the *tupunus* communicates to the spirit of the stone his desire, and its magical power accomplishes that. *Kaha* Katmatem added that the requests put forward during *temahwa* do not go directly to the spirits. The requests, and anything else that happened in the *nakamal* that day, are sent to the setting Sun, who then delivers this report to the spirits. Though not tied to the same geographical features as in the Nafe area, the Sun and its movements are still a very important aspect of *temahwa* across Tanna.

#### Tracking the Sun’s yearly progression with trees and landmarks

Since Kalpapen’s first *temahwa*, Tanna has seen regular day-night cycles in periods conducive to human work and health. However, the Sun’s position and length of time in the sky change throughout the year and there are a number of different strategies on Tanna that are used to track these gradual changes. One of these strategies involves tracking the progression of the position of sunsets over the course of the year in relation to a set of familiar trees, helping to keep track of and solstices as a kind of living Stonehenge.

It seems that only a small number of elders continue to practice this tradition, or remember it well enough to discuss it. However, elders in the Netwar, Newai, and Nanunata languages all described how they track the Sun using trees in similar ways. The viewing angle is most often centered on one’s preferred spot in the *nakamal*, where he would go sit down after having had his kava. As kava is consumed before sunset, one can sit quietly after having made his *temahwa* and observe the point of the horizon where the Sun is setting. He would then mentally mark specific trees aligned with this point, and over the course of the year would then remember the succession of trees that match the sunset at various weeks or months, especially remembering those that represent the two endpoints, after which the setting position reverses, representing the two soltices.

The time of the year surrounding the winter solstice is called *nian rerparep* (lit. ‘short days’), or alternatively the more poetic term *nowa nalulu* (lit. ‘fruit of *nalulu*,’ *Plerandra tannae* (A.C. Sm. & B.C. Stone) G.M. Plunkett, Lowry and Frodin) in reference to the season during which this plant species makes its fruits. It is the colder time of year and the time of harvest, feasting, and *kastom* ceremonies. Around the time of the summer solstice, the season is called *nian apom* (lit. ‘long days’), or in a more descriptive fashion as *nian taha nasuman* (lit. ‘days of work in the garden’). It is the time of planting gardens, progressively less feasting, and many taboos which aim to ensure the spirits can do their work in the garden to ensure a plentiful crop. The solstices themselves are called *met ramleleg* (lit. ‘the Sun returns’) and especially the winter solstice is observed as it marks the time when the colder period of the year is about to finish and the days of readying the gardens are coming.

Apart from using living trees, a parallel Sun and season-tracking strategy involves named geographical features and *kastom* roads. As the Sun and Moon make their way across the sky, they are considered to be walking their *suatu* ‘roads.’ These roads have counterparts on the island in both *kastom* roads used by people and individual places, such as stones or caves, that often bear solar or lunar toponyms. Because the Sun’s path throughout the sky shifts throughout the year, it does not follow a single path. This is reflected in local views, where watching the Sun in relation to these *kastom* roads and places is one strategy used to track the seasons in order to properly tend to the gardens. More specifically, the Sun is said to walk its own roads in the sky. When the days are longer, the road is known as *nekaugan apom* ‘the long empty space’, While during shorter days, it walks walks *nekaugan rerparep* ‘the short empty space.’ One terrestrial *kastom* road strongly associated with the Sun is one of the main east-west arteries across the island, *Nukulua*. When one stands on this road and sees the Sun both rising over and setting over the same road, it is the time of *nian apom* and the time of gardens. During the time of *nian rerparep*, the Sun does not follow *Nukulua*, but rather a path to the north of this road.

Other places on the road of the sun and Moon include *Lémetmawuk* ‘the place of Sun and Moon,’ a cave near the Tanna airport through which the Sun and Moon both pass on their journeys. A similarly named sacred stone sits near Lamkail village, and is called *Metmawuk* ‘Sun and Moon.’ This stone has a clearer association with the practice of tracking the seasons using solar toponyms. It is considered by some to be the starting point of the yearly journey of the Sun through the sky. Later during its route (i.e., during the winter solstice), the Sun takes *khakel* ‘planting stick’ and comes back, passing through the village of Lenus at the time of month called *nus*, in which the yam vines flower. Then the Sun reaches the *Metmawuk* stone and starts the year over. A Nahual-language analogue to this stone is *Imelmakua* ‘house of the Sun and Moon,’ a set of two sacred stones—a white one for the Sun and a black one for the Moon.

#### The Sun’s influence on the layout of gardens

The layout of gardens and the location of other plantings on Tanna are influenced significantly by considerations of exposure from the Sun. In many parts of Tanna, people live on ridges and make gardens both on the ridge itself, and also downslope and sometimes deep along creeks. Ridge gardens are called *nasuman iles* ‘upper gardens’ in Netwar, and creek gardens are *nasuman lahau* ‘lower gardens.’ Factors that influenc what crops are planted in each of these gardens include sunlight, moisture, winds, and accessibility. Some crops are essentially obligately planted either on the ridge or along creeks, but others can be planted more or less anywhere based on considerations of food security (e.g., quick accessibility on ridges, or greater protection from harsh weather downslope). *Kepwia* [*Alocasia macrorrhizos* (L.) G. Don, known locally as ‘taro Fiji’) prefers the shady places of lower gardens, but can also be planted in the upper gardens. This crop is often planted in lower gardens to ensure that it is available in case of cyclone damage. *Nepen ituga* and *saina* (two short varieties of banana considered to originate from abroad) can be planted in upper gardens for convenience, but it is often better to plant them also on the lower gardens, as they are short, resist the wind well, and keep their fruit even if a cyclone passes through. *Nuw* ‘yam’ is easily destroyed when there is too much rain or too much sun, so it is important to choose the right place between the ridge and the creek where conditions are optimal. *Neté* ‘taro’ (*Colocasia esculenta* (L.) Schott) can be planted in upper or lower gardens, but often thrives better in lower gardens. By contrast, *Kep* (a kind of taro without large tubers) is planted exclusively in the wettest places of the bottom of the creek in the lower gardens. It is appreciated for its tender and flavorful leaves, which are cooked with coconut milk and consumed alone or as a side dish. *Nién* ‘coconut’ is planted almost exclusively in sunny places higher in the gardens.

**Fig 5:**
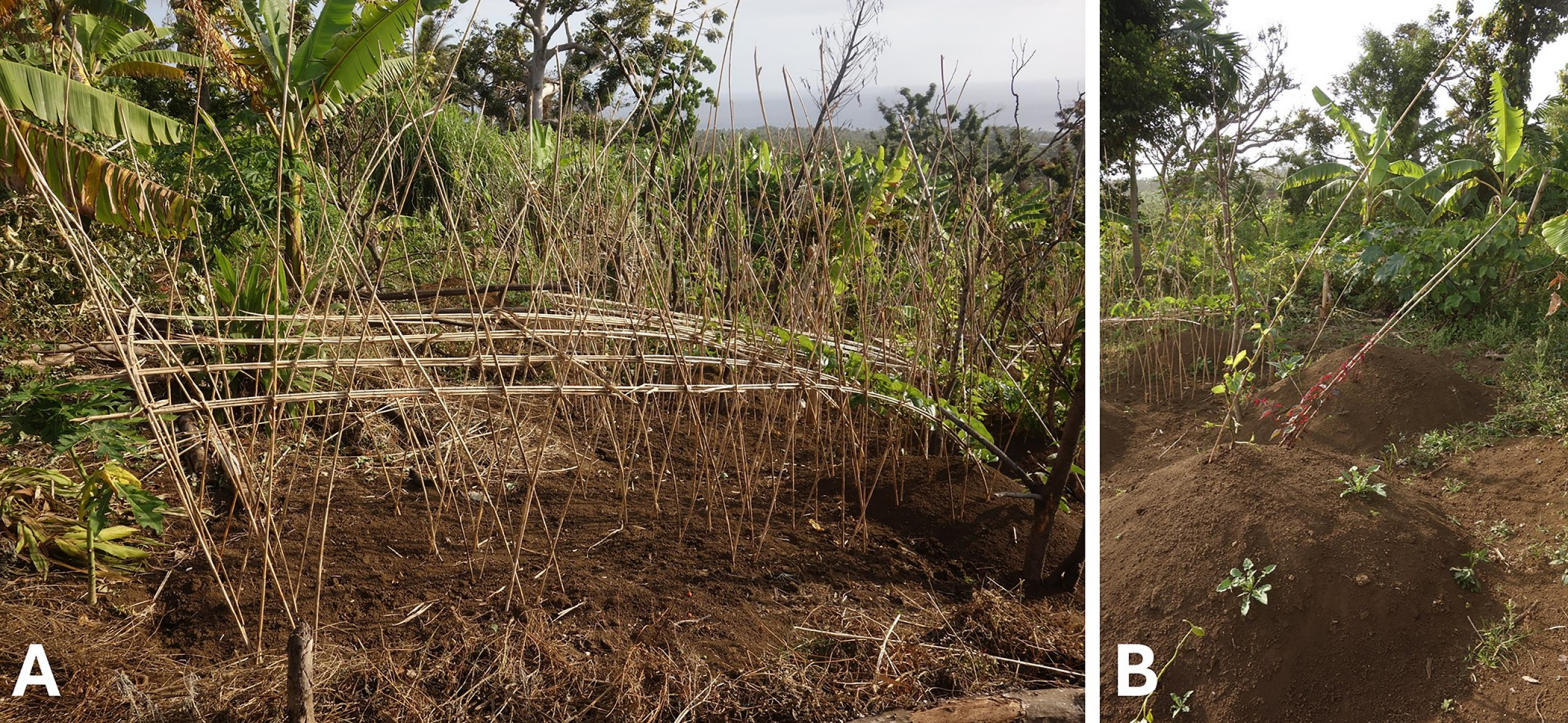
Garden yam supports. A: *kamé piagen*. B: *kamo sit*. Photos by Dominik M. Ramík.

Yams are planted with their “head” (the part of the yam which protrudes from the *tow*, the mound) towards the morning Sun as they will grow better this way. Larger yams are trained using a complex system of wild cane supports called *kamé piagen*, while the simpler *kamo sit* can be used to train smaller varieties (see Fig 5). Taro, on the other hand, is planted in a small pit and the dug-out earth is heaped towards the setting Sun, which will protect them and make them grow better. When planting trees, it is better to plant them in the open, not under the shade of other trees. Such newly planted trees need both sunshine and rain, so they are generally planted in cleared places. Coconut trees, for example, must be planted in a cleared place. Otherwise, they will not produce many fruits. Some trees, however, require more shade to become established, and thus planting *napek* ‘banyan’ (*Ficus prolixa* G. Forst.) is viewed as a very good way to gives “freshness” to the small trees that grow beneath its shade.

Outside of the Netwar zone, in the Nafe language area, some common gardening considerations are as follows. Yams are grown closer to the coast because there is more Sun there and it is warmer. Because of this, the *tupanas* for yam come from coastal tribes. By contrast, taro prefers colder weather that is not as sunny, so it is better to grow taro in higher areas, and the *tupanas* for taro come from bush (inland) tribes. Yams are planted and grown in mounds (one yam in each mound), using the tip of a previously harvested yam, which then produces a new tuber. The old yam portion is not planted into the top of the mound but on its west-facing side. That way, the Sun will heat up the east-facing side all morning, helping to break down the old tuber, and as it breaks down, it helps to “feed” the newly developing tuber that is growing beneath it. This strategy will help the new tuber to grow very large. Once the shoots emerge, a trellis is constructed to support the yam vines, and this is oriented so that it leads the vines away from the wind, helping to prevent the wind damage to both the vines and the trellis. Kava and/or taro plants may be planted between the yam mounds, but only after the yams are planted because this later period of the year is wetter, and kava and taro plants prefer wetter conditions.

#### The Sun and *kastom* roads in architecture

**Fig 6:**
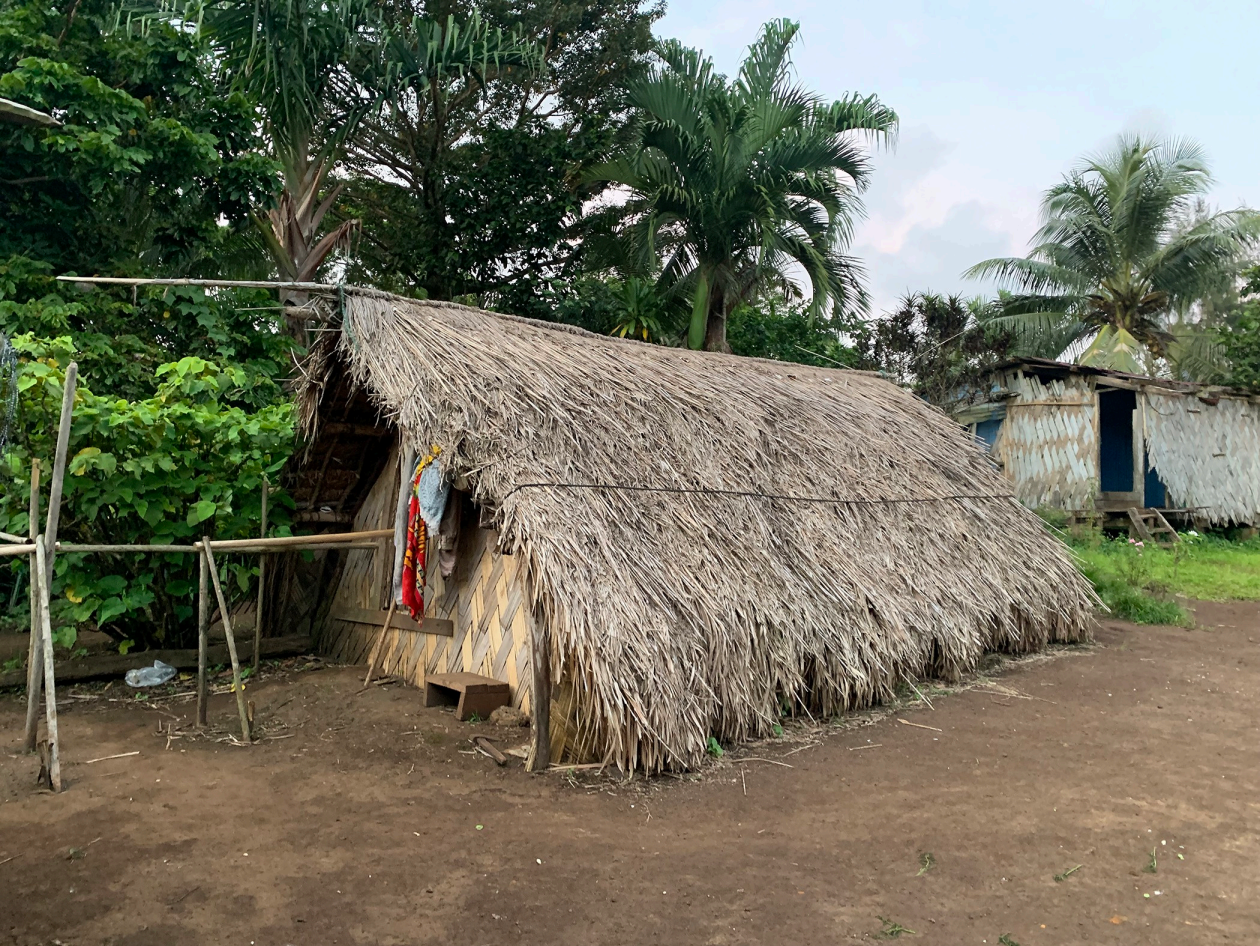
Nimah le Tən. A traditional house of north Tanna. Photo by Neal Kelso.

In determining how to orient traditional houses (Fig 6) on Tanna, the Sun is often at least one part of the consideration, but there are diverse rationales for whether the primary axis should be north-south or east-west. Ultimately practices are very local, determined in part by geography and the traditions of a particular village. In villages that orient their houses with the long axis north to south, the rationale is often that the Sun rests on both halves of the roof for a good portion of the day, burning off the moisture and thus keeping the roof thatching dry and rot-free. In addition, the primary wind from cyclones (from the north-west) will pass through the openings of the end walls rather than buffeting the roof, making it more likely that they will remain standing during a strong storm. In many villages, the most important consideration may actually be the direction of the primary *suatu* in the area. The rationale involves the *ierames* spirits, which walk along such roads. If the openings of the house follow the same direction as the road, then these spirits can pass through villages unhindered, but if the orientation were perpendicular to the road, the *ierames* would get stuck and accumulate in the village until harm came to the villagers.

In the Nanunata language area, on the eastern coast, houses are generally oriented east-west. The logic is that this orientation allows the Sun to shine directly into the house in the morning to warm it up. In the Newai language area, houses are also most often oriented east-west. In the village of Lenapos, it is said the Sun “walks” the *kalwau*, the ridge beam at the apex of a house following its major axis. In addition, the winds coming from Mt. Iasur carry acid rain and ash, which would cause rapid and uneven damage to the eastern side of roof if the house were oriented north-south.

A group of elders from the village of Enfitana gave a more detailed account of the reasoning behind house orientation and the internal architecture of houses. The east-west orientation of houses is again determined by the path of the Sun. The most important *suatu* in this part of Tanna (through the entire Letakren area, from roughly Lenakel up to Lenapos) is Nukulua, which follows roughly east-west and goes from the eastern to the western shore of Tanna, paralleling the movement of the Sun. The ridge beam is understood to be a reproduction of Nukulua. The smaller vertical rafters on the sides of the house are called *nowanetan* (lit. ‘pieces of ground’), as they are anchored in the ground to support the roof. They symbolize the slopes of the creeks going east-west, the house itself representing a geographical ridge. Smaller horizontal rafters (or battens), called *kalwau lahau* ‘lower beams’ symbolize another series of concentric ring roads, which encircle Tanna at various elevations. Kwotarén is the lowest of these traditional roads, passing along the seashore around most of Tanna. Naniarap is another ring road found at a higher elevation. Matakaiu is higher yet, and Nemanahnep is the uppermost of these kastom roads, found near the summit of high-elevation sites. Thus, a traditional house of Tanna, *nima le Ten*, itself represents a map of Tanna. This is not to neglect the fact that there are dozens of traditional roads connecting the nakamals of Tanna, but the roads named here are among the most well-known.

In the Nafe language area, traditional houses, now called “cyclone houses,” are oriented east-west, with the door facing east. This allows a fresh breeze to enter, and it also allows the strong morning Sun to clean out the house. Further, since these houses are built intentionally for the thatched roofs (which extend to the ground) to withstand strong cyclones, this orientation keeps the winds from breaking the weaker end walls and blowing aggressively through the house while people are sheltering.

Another relationship between the Sun and architecture can be found in Tanna’s “Sun houses,” in which any rituals to influence the Sun are supposed to take place. In the north, they take the form of a small round house, which is only rarely seen on the island today. These houses are named *nima tikiskis* in the Naka language, after the bird *tikiskis* (*Rhipidura albiscapa* [Gould, 1840], grey fantail). The shape of the house is perhaps reminiscent of an inverted *tikiskis* nest, which is conical in shape. This bird is also generally associated with both the Sun and whirlwinds, in addition to being considered to be the mother of all birds. Thus, *nima tikiskis asim*, “the sacred house of *tikiskis*,” is the “Sun house” in which Koden Dick, *tɨpunɨs* for the north wind, performs weather magic in his village of Ikampala o Luwətu Iken. They must be built in a single day during *nian os*, the time of harvest, while observing the rites of *natuakaman*, which includes fasting and abstinence. Inside, only two special kinds of wood may be burned: *nɨkfitu* [*Lepidocupania brackenridgei* (A. Gray) Radlk.] and *nɨkaɨp* (*Geissois denhamii* Seem). By observing these and other rites, the man working inside the house can perform rituals to influence both the Sun and weather, sometimes supported by other local *tɨpunɨs* working outside. If sunlight ever reaches the inside of this house through its roof, the entire house must be destroyed and remade, though the sacred ashes accumulated on the ground may not be touched. Sun houses were made and used across the island in the recent past, and another made by *Kaha* Iahwa can be seen in Lamanamileg. It is small like the *nima tikiskis asim* of the north, but is not itself round.

## Discussion

In both Tanna’s *nagé* legends and in present understanding, the Sun is a living, personified being of great importance to the people of the island. In the creation cycle, Wughin and Kalpapen are clearly intent on providing the world with adequate light for its inhabitants, making various attempts until ultimately arriving at the day/night cycle we have today. This process also resulted in the creation of the *temahwa*, in which the Sun assists human *tupunus* in getting their requests to the spirits. In later stories, the Sun is both protagonist and antagonist in its relationships with other beings—it is simultaneously parent to the gardens and a burning force with the power to destroy them.

The language of the *nagé* legends itself is also informative of the Sun’s entanglements with other life. The word for ‘man’ or ‘person,’ *ieramim*, is constantly interchanged with specific words like mountains, trees, animals, humans, spirits, and the Sun. This underlines the sweeping notion that all beings in Tanna cosmology are living people. They have their spirit and personality and interact with other people, both human and non-human. These interactions are clearly seen in the *nagé*, as the Sun works with and against plants, lizards, stone-men, and spirits, both in the past and at present.

Although Wughin’s early attempt at providing light with the *nekawuk* tree was unsuccessful, today the relationship between the Sun and plants is key to maintaining livelihoods on Tanna. People on Tanna lead biodiversity-dependent lives, with plants being the primary part of their diets, architecture, medicines, crafts, and more. The Sun’s role in these practices is evident in the results of the present paper: trees are used to track the Sun’s yearly progression, houses made of plants represent the Sun’s movements, flowers are used to mark the passage of time, and the placement and layout of gardens depends greatly on considerations of sunlight.

These entanglements are in fact even stronger. Seasonal time is reckoned not only by the Sun, but also by the Moon, winds, plants, and animals in a holistic system used to maintain human and ecosystem health [35, 36]. Many “calendar plants” are now only remembered by the oldest generations, but those that guide the agricultural cycle—among the most important activities on the island—are more widely known. The phenology of certain plant species can inform people of when the sunlight is becoming too long and strong, both for people and for plants. In the cases when the Sun causes or will soon cause damage despite the warnings of plants, both medicine and magic are available to the people of Tanna for prevention and healing. As in the *nagé*, the Sun today can simultaneously be a positive force for people and cause harm.

## Conclusion

On Tanna, the Sun is not a distant celestial body, but rather an active player in the biosocial, multispecies cultural landscape of the island. It provides information—either directly or transmitted through plants or the land—guiding people in time-reckoning, agriculture, architecture, and more. Cultural practices and ultimately the landscape itself are shaped by the relationships that *ieramim*, be they human or non-human, have with the Sun.

These solar relationships, in their present state, are dependent on the maintenance of local languages, cosmology, and epistemology. Storytelling, including but not limited to *nagé* legends, passes on expansive ideas about the nature of life to subsequent generations. While people on Tanna will always be affected by the Sun, the details of those effects are changing due both to cultural and climatic shifts. Conscientious support for Indigenous languages, stories, lands, and other cultural practices are paramount as Indigenous peoples seek both to maintain their traditional ways and to adapt to a rapidly changing world.

After the devastation to Tanna wrought by Cyclone Pam in 2015 [37], there was renewed interest across the island in sharing elders’ knowledge of how to build traditional houses. These houses survived the storm more intact than four-walled houses, whether wooden or concrete. In an effort to pass on traditional knowledge such as this to younger generations, local cultural specialist Jean-Pascal Wahe organized Tanna’s first *Kastom Skul* (‘Custom School’) in Iatukwei village in 2018. A group of knowledgeable elders—who are acutely aware of both cultural and climatic shifts—convened to transmit knowledge to younger community members, not only about construction of cyclone houses, but also on tying knots, weaving, ropemaking, cooking, and flute music. A number of communities across the island have also been active in recording environmental knowledge for dissemination to young people in Talking Dictionaries and a print manual of plant uses and lore in the province of Tafea. A new version of the dictionaries is currently in development, with the intent that users can create linguistic posters, document songs and stories, and more easily teach others how to maintain the project’s goals. A manual of useful plants is also nearing completion and will be disseminated throughout the province in early 2025 [38]. Though things are changing, the people of Tanna are working to ensure that such change is to their benefit, maintaining evolving traditions while also adopting and adapting to new technologies and cosmologies.

## Supporting information

S1 File Inclusivity in global research. (DOCX)
